# The Relocation Problem of Field Calibrated Low-Cost Sensor Systems in Air Quality Monitoring: A Sampling Bias

**DOI:** 10.3390/s20216198

**Published:** 2020-10-30

**Authors:** Georgi Tancev, Céline Pascale

**Affiliations:** Swiss Federal Institute of Metrology, 3084 Bern, Switzerland; celine.pascale@metas.ch

**Keywords:** air quality monitoring, calibration of chemical sensors, low-cost sensors, machine learning algorithms, sampling bias

## Abstract

This publication revises the deteriorated performance of field calibrated low-cost sensor systems after spatial and temporal relocation, which is often reported for air quality monitoring devices that use machine learning models as part of their software to compensate for cross-sensitivities or interferences with environmental parameters. The cause of this relocation problem and its relationship to the chosen algorithm is elucidated using published experimental data in combination with techniques from data science. Thus, the origin is traced back to insufficient sampling of data that is used for calibration followed by the incorporation of bias into models. Biases often stem from non-representative data and are a common problem in machine learning, and more generally in artificial intelligence, and as such a rising concern. Finally, bias is believed to be partly reducible in this specific application by using balanced data sets generated in well-controlled laboratory experiments, although not trivial due to the need for infrastructure and professional competence.

## 1. Introduction

The effects of air pollution are well known and the health impact is massive, and with the increasing public awareness about the adverse health effects of air pollution, e.g., by fossil-fuel combustion, the urge to monitor and regulate the amount of hazardous gases or particulate matter (PM) is becoming even more important [1,2,3,4]. Air quality monitoring (AQM) stations are expensive and therefore coarsely distributed across cities. Increased traffic and unfavorable meteorological conditions can quickly lead to local exceedance of the exposure that will not be noticed, so higher spatial resolution would be desirable [5,6].

More and more start-ups are entering the AQM market with novel low-cost sensor systems connected to the internet; some emerged from know-how in classical analytical chemistry, others from expertise in the Internet of Things [7,8]. The latter put too much trust in the used hardware, e.g., sensors, even though these often suffer from low performance due to interferences/cross-sensitivities, drifts, and large unit-to-unit variability, as a lot of research on low-cost sensors and devices has shown [9,10,11,12,13,14,15,16,17,18].

The two sensing principles which low-cost gas sensors are based on are reduction–oxidation reactions in electrolytic cells and adsorption–desorption reactions on metal oxide surfaces, both with their own issues [7]. For instance, electrochemical sensors are reported to be faster and less prone to drift due to aging, but also less sensitive than metal oxide sensors [7]. PM sensors are usually based on light scattering and the most significant interfering variable relates to water, as they appear to overestimate PM mass under high relative humidity [7,16].

Current academic discussion revolves around correcting for interfering variables and improving sensor performance by using mappings, known as calibration functions, obtained from different machine learning (ML) algorithms, e.g., neural network (NN), random forest (RF), or (regularized) linear regression (LR) [8,9,19]. This is a standard regression task and it is still a point of discussion which of these algorithms are most suitable for that kind of application as some algorithms are better than others at coping with non-linear systems [8,20,21]. Due to the contributions of preprocessing procedures such as data normalization and outlier removal, and distinct sensor models originating from a variety of manufacturers combined with the aforementioned unit-to-unit variability of sensors, comparison of results across publications becomes tedious [8,15].

Lastly, sampling of data for calibration, e.g., in the field, in the laboratory, or both, is often a topic of discussion. Field calibration of low-cost sensors, i.e., collocation with reference instruments, enables more combinations of variables at lower price but often leads to relocation problems, both spatial and temporal [9,22]. More precisely, the performance of a low-cost sensor system calibrated with measurements from one location decreases after being moved to another location, in addition to the decrease of performance over time at the same location.

Since these problems are often reported but not necessarily always understood, it is worth revising them [22]. By using published data sets, the aim of this work was to identify the origin of relocation artefacts, investigate their dependence on the different algorithms by inspecting models and joint probability distributions of input and output variables with methods from data science, and discuss potential solutions.

## 2. Methods

In the following, the motivation for alternative data analysis methods (together with the overall workflow) is illustrated with an emphasis on LR, as it is familiar to most readers. However, the situation is analogous for other ML algorithms such as NN or RF; the reader is encouraged to consult fundamental statistics and ML literature for more details and explanations of all methods applied in this analysis [20,21,22,23].

### 2.1. Workflow

Suppose a scientist wants to predict the influence of a set of independent variables (inputs, features), stored as a matrix X, on a set of continuous dependent variables (outputs), also stored as a vector or matrix y, with LR. In many cases, the scientist might know already from the literature which variables to include in the analysis, although not necessarily all of them. Alternatively, it might be desired to include power/interaction terms to account for non-linearity (an approach called basis expansion) or new variables in the model, which might increase the number of variables heavily. To study the relative contributions, it is helpful to scale all variables to a common range, e.g., by subtracting the mean and dividing by the standard deviation, a procedure termed standardization. (On a side note, this also facilitates numerical computing.)

In classical statistics, the scientist would fit models and either discard or retain variables depending on the *p*-values of their parameters β. The goal is to control model complexity and to obtain a model that generalizes well. It should be neither too simple (high bias, underfitting) nor too complex (high variance, overfitting); this is called bias–variance trade-off. Such an approach can be tedious if no prior knowledge is available, as many potential configurations of variables have to be assessed.

In LR, the optimal parameters are obtained by minimizing some loss function L, e.g., the least squares error (here written in vector notation):

L(β) = (y − Xβ)^T^(y − Xβ) (1)

There is a closed form solution for this optimization problem stated in Equation (1), which can be obtained by computing the derivative with respect to β and setting it equal to zero, resulting in Equation (2):

β = (X^T^X)^−1^X^T^y (2)

In the case of a multi-output problem, β is a matrix. If the number of variables becomes very large, many of them can end up in the model, although the researcher is probably only interested in the most important contributions. Furthermore, the inversion that is present in the closed form solution does not exist if the number of variables is larger than the number of samples. In these scenarios, methods from ML can help.

More precisely, a penalty term for the parameter vector can be introduced in the objective function in order to control model complexity, for example the squared Euclidean norm:

L(β) = (y − Xβ)^T^(y − Xβ) + α(β^T^β) (3)

This approach is called regularization and the (regularization) parameter α ≥ 0 regulates it. Now, for α = 0, the loss function reduces again to the least squares error, but for larger values, the algorithm optimizes the choice of β such that only the most relevant variables are retained. The penalty term above is essentially the L_2_-norm of the parameter vector, which is the reason why this method is termed L_2_-regularization (but it is also known as Ridge regression). The solution is:

β = (X^T^X + αI)^−1^X^T^y (4)

For every value of the regularization parameter α, another solution for β is obtained, which is why α is also called hyperparameter (HP). The optimal value for α is not known a priori, though, and the extent of HP optimization has an influence on the resulting model performance. However, jointly optimizing for α and β would result in α = 0 and the least squares solution, so this is not a viable approach.

Instead, a discrete set of values (a grid) for α is constructed; usually, the spacing between the individual values is chosen to be evenly on a logarithmic scale to obtain higher resolution for small values of α. Next, for each value of α, the training data set is sliced into k folds, whereas k − 1 folds are used for training, i.e., computing β, and the last fold is used for validation (the literature proposes values in the range of three to ten for k). The average error from all validation folds is computed for every value of α, and the α corresponding to the lowest average error is considered optimal. These techniques are called grid search (GS) and cross-validation.

As a result, a model with several coefficients much smaller than others (i.e., irrelevant variables) is obtained; and while not necessary, removing these variables makes it more compact and can speed up predictions in some situations (model pruning). It is reasonable to start with a large number of model parameters and proceed to prune models once a good benchmark performance has been found.

Usually, the data set is shuffled and split into training and test set in the beginning of the analysis. The training set is used for training and validation, whereas the test set is only used for the evaluation of the final model. Since some methods (such as shuffling) are based on random number generators, it is also important to fix them. Otherwise, the composition of training and test set changes with every run, and comparison of results becomes impossible. This is particularly true in the presence of outliers, which should be removed in advance.

Occasionally, it might be useful to study the global structure of a data set in order to detect outliers or other patterns, which can be achieved with dimensionality reduction methods such as principal component (PC) analysis. Such an analysis reduces the number of variables that are correlated to each other into fewer independent variables, enabling visualization of data in two or three dimensions.

Once a good model has been obtained, it is recommended to analyze and interpret it with additional model inspection techniques. The model performance as a function of number of samples (learning curve) can reveal if enough data has been collected. Additionally, the model performance as a function of regularization parameter (validation curve) helps to assess whether the chosen grid interval is large enough.

Even though the importance of variables is easily interpreted in LR, there are other ML algorithms in which this is not achieved so easily, and hence alternatives have been developed for doing so. One such method computes the distortion of the model (i.e., average decrease in performance) upon permutation of values (permutation feature importance). A relevant feature will lead to worse predictions if its values are permuted. Additionally, a partial dependence plot visualizes the average effect a feature has on an output variable.

It should be apparent by now that the workflow, described here in a linear fashion, is actually iterative in nature. As a final remark is worth mentioning that, although only closed form solutions have been presented above, gradient-based iterative numerical methods for minimizing the least squares loss function exist as well; they are particularly suited for larger data sets because computations can be performed in less time, but an extensive explanation thereof goes beyond the scope of this introduction.

### 2.2. Procedures

The air quality data set from De Vito et al., collected on a main street in the center of an Italian city characterized by heavy car traffic, is used to examine the influence of field calibration and different algorithms [24]. Moreover, the aim is to measure gas compounds in air using low-cost sensors and to correct potential but unknown cross-sensitivities as well as environmental interferences.

The data set consists of hourly sensor signals (inputs) of CO, NO_x_, NO_2_, O_3_, temperature (T), and absolute humidity (AH), and reference signals (outputs) of CO, NO_x_, NO_2_, and C_6_H_6_ as time series over the course of one year. Note that there is neither a sensor for C_6_H_6_ or pressure (P), nor a reference for O_3_. De Vito et al. advocate for a half-yearly recalibration interval [24], so only the data collected during the first half year are used (N = 4400).

Analysis and modeling is performed in Python using the open-source libraries Pandas and Scikit-Learn [25,26]; the former is a library for data manipulation, whereas the latter offers a variety of ML algorithm implementations and techniques for preprocessing as well as model interpretation. The regression algorithms evaluated in this work are NN, RF, and LR, each as one single model with all four outputs combined for the sake of simplicity [20,21].

Raw data are preprocessed before analysis, i.e., standard scaling to zero mean and unit variance, removing instances with missing values (ΔN = 1300; mainly time points with missing reference values for CO, NO_x_, and NO_2_), removing outliers with isolation forest (ΔN = 400) [27], and shuffling (N = 2700).

The data set is partitioned into training set (70%) and test set (30%). Due to bias–variance trade-off, algorithm HPs such as regularization parameter α or maximum depth of trees are optimized via cross-validation using 5-fold GS with negative mean squared error (MSE) loss to control model complexity. To guarantee reproducibility and repeatability, random number generators have been fixed for every single method.

Lastly, the models are evaluated and their behavior is inspected by computing learning and validation curves, permutation feature importance, and partial dependence [21]. The relevant evaluation metrics are MSE, coefficient of determination (R^2^ score), and the agreement/slope (*ρ*) between ground truth and predictions, all computed from test set data and averaged over all output variables.

### 2.3. Algorithms

#### 2.3.1. Neural Network

An NN is a model archetype whose working principle is conceptually derived from biological neurons. It consists of nodes (“neurons”, hidden units, latent variables) and the arrangement of connections between nodes is called topology. Usually, nodes are arranged in layers, receiving information only from previous layers and transmitting information to consecutive layers (feed-forward). In mathematical terms, the formulation is given in:

Z_t+1_ = h(W_t_Z_t_ + b_t_) (5)

In Equation (5), Z_t_ is the vector with values (“information”) from the current layer t, W_t_ is a matrix with the weights connecting the two neighboring layers, b_t_ is a vector with constants, h is termed activation function, and Z_t+1_ is the vector with values of the next layer t + 1. The algorithm to “train” NNs is called backpropagation [20,21]. NNs can model arbitrarily complex functions. However, they are often referred to as “black-boxes”, because their internals are opaque and it is not clear which inputs affect which outputs in which direction [28]. This is the reason why they benefit the most from additional model inspection techniques.

In this work, the initial NN consists of three hidden layers with fifteen latent variables (nodes) each and rectifier activation function. Via GS, L_2_-regularization parameter α is optimized within the discrete set of 51 logarithmically spaced values between 10^−2^ and 10^2^. Pruning is performed to obtain a compact topology by sequentially removing hidden layers and latent variables unless performance decreases.

#### 2.3.2. Random Forest

An RF is a collection of decision trees (hence a forest) that performs binary splits on data points, i.e., answers with yes or no on each split. A decision tree is characterized by its depth (the number of decisions it is allowed to perform), whereas each tree in the RF can have a different depth. Every tree is trained with a random subset of data by resampling training data with replacement. In the computation of a split, only a random subset of variables is considered, and the variable (and its value) are selected according to some optimality criterion, e.g., sum of squares. As a consequence, every tree will be composed of slightly different decisions and provide a different prediction value; the effective prediction is an average across all output values from all trees [21].

Here, the RF consists of 1000 trees. As HP, the maximum depth of a tree is restricted to all integer values between 4 and 12 and optimized via GS.

#### 2.3.3. Linear Regression

Much has been written about LR in the previous section already. One important remark is that, besides L_2_-regularization, there are two other important regularization alternatives, namely L_1_-regularization (least absolute shrinkage and selection operator, or simply Lasso) and a combination of both L_1_-/L_2_-regularization (Elastic Net). The difference lies in the handling of correlated features, i.e., variables that contain the same information. Whereas the L_1_-norm retains only one variable in subsets of correlated variables in a random fashion (and leads to so-called sparse solutions), the L_2_-version retains all variables in a set of correlated variables [20,21].

Thus, it should be stated that the L_2_-regularized version of LR is used here. In addition, basis expansion up to a power of two is introduced for all input variables before model building to account for potential non-linearity. Via GS, regularization parameter α is optimized within the discrete set of 51 logarithmically spaced values between 10^−2^ and 10^2^.

## 3. Results and Discussion

**Figure 1:**
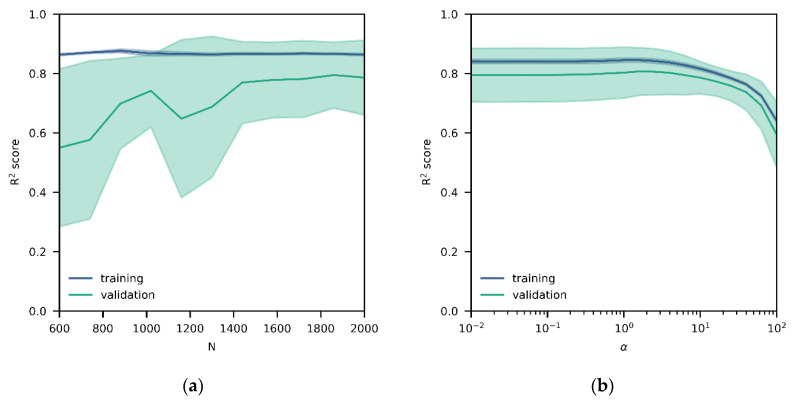
(**a**) Learning curve for training (blue) and validation (green) data sets for the final neural network (NN) model (with mean and standard deviation); validation set performance increases with increasing sample size N and flattens above 1600 instances; (**b**) validation curve for training (blue) and validation (green) data sets for the final NN model (with mean and standard deviation); validation set performance decreases with increasing α, i.e., a too simplified model, but is fairly stable below values of α = 1.

In the following, only detailed results for the NN model development are presented to demonstrate the added value of model inspection, and the RF and LR results are provided as supplementary material. Figure 1 shows the learning and the validation curves of the NN model with respect to the R^2^ score after pruning (final topology consists of one hidden layer with ten latent variables). The blue line is the expectation in training set performance of the five folds, whereas the blue area is an approximation to the variability, i.e., standard deviation, within all folds; the same information is visualized in green for the validation set. A higher amount of training data increases the R^2^ score for the validation data set, but this performance increase flattens above 1600 instances (about two months of hourly measurements), indicating that this a sufficient quantity of training data (Figure 1a). Surprisingly, there is a systematic bias between the training and validation set performances despite shuffling, which might be caused by the inherent noise in the measurements; an equivalent pattern is observed in the validation curve, in which R^2^ decreases with increasing α, i.e., a too simplified model (Figure 1b). The same behavior is observed independent of the algorithm (Figures S1 and S2).

Table 1 summarizes the performance of all ML models. For the same data set, insignificant differences in algorithm performance are obtained, which is backed up by the academic ML literature as the influence of algorithms on predictions has been reported to be marginal for a high amount of data [29]. The meaning of “high” is surely context dependent, but since it is known that sensor-reference relationships are mostly linear, it is reasonable to not find any major performance differences between algorithms. The result should still not be interpreted as an equivalence of ML algorithms [20,21]. For example, non-parametric algorithms like RF cope better with non-normally distributed data but fail to extrapolate. An advantage of NNs and RFs over LR is the possibility to construct non-linear functions without explicit basis expansion. Furthermore, LR and RF models can be interpreted without additional methods, which is not true for NNs. Lastly, there are also differences in computational complexity, i.e., the time needed to find a solution, although irrelevant with this small amount of data.

**Figure 2:**
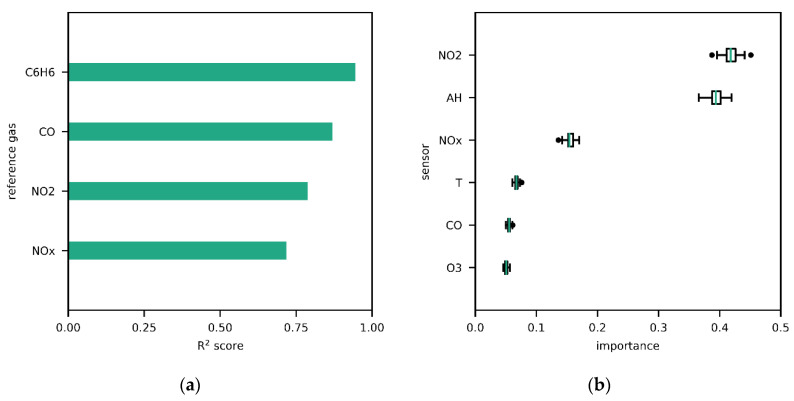
(**a**) Model performance of the NN with respect to individual references; (**b**) permutation feature importance of all features in the NN model.

By inspecting the different models, the spatial relocation problem becomes evident. In Figure 2a (also Figures S3a and S4a), R^2^ for the individual outputs is plotted. Surprisingly, C_6_H_6_ has the best prediction performance although without corresponding sensor. According to permutation feature importance, only a few sensors appear to be relevant for the NN model, but knowledge of the CO sensor signal is seemingly redundant even though there is an output, i.e., reference values, for CO (Figure 2b). However, every input paired with an output should be considered important, unless a sensor is not working properly. Relying only on small subsets of sensors is a property that the three models have in common (Figures S3b and S4b).

**Figure 3:**
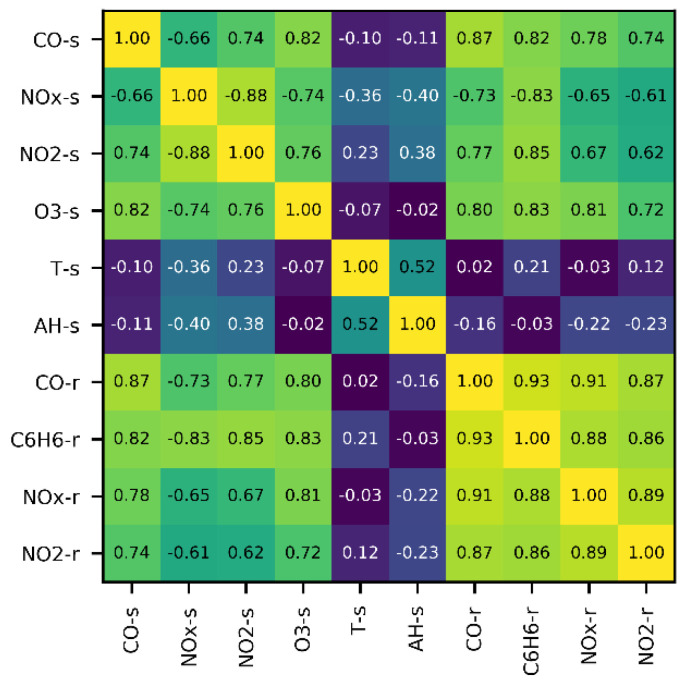
Spearman rank correlation matrix of sensor (s) and reference (r) signals.

When examining partial dependence plots, many of them being linear, it can be noticed that one single gas sensor could technically “measure” all outputs (Figures S5–S7). (Note that permutation importance values and slopes of partial dependences are interrelated, i.e., the magnitude of the slope is proportional to the importance value.) This is only possible because outputs are correlated, which becomes evident when analyzing the Spearman rank correlation matrix (Figure 3), as all input and output variables are indeed heavily coupled. Note that absolute correlation values between sensors and their corresponding references are above 0.6, but so are correlations with all other references.

From a chemical point of view, the strong relationship between pollutants is not surprising at all; it is as if there was one single machine burning fuel or coal with a constant reaction mechanism (fixed stoichiometry) placed next to the AQM system. Although this is likely an extreme case, it shows that the origin of the relocation problem of field calibrated sensor systems is independent of the used algorithm. Still, one could argue that an RF should distribute importance evenly on all variables since only a randomly sampled subset is considered for the computation of a split in a tree. However, L_2_-regularized solutions are known to not deliver sparse solutions either (as opposed to L_1_-regularization); and in fact, partial dependences are correlated and mostly non-zero, so the problem really lies in the data. A sensor subset that minimizes the MSE the most without needing too many overall weights (due to L_2_-regularization in the case of NN) is chosen to predict all other references as well, in this instance apparently NO_2_ and AH. Since sensors can act as substitutes for each other due to correlations in reference data, assessing their functionality solely by feature importance is challenging.

**Figure 4:**
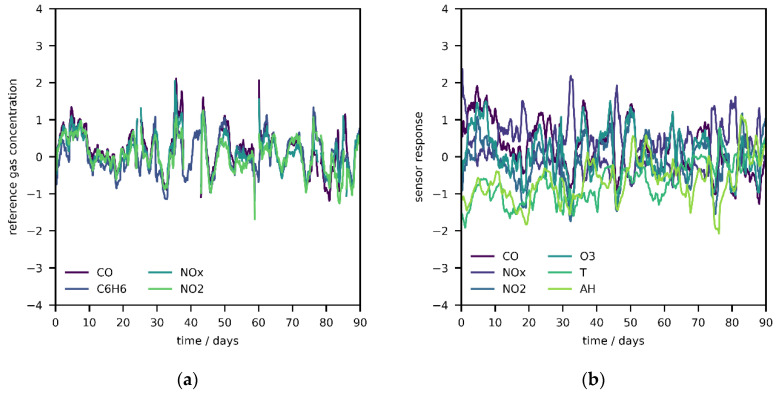
(**a**) Time series section of standardized reference signals smoothed with moving average of 24 h windows; (**b**) standardized sensor signals smoothed with moving average of 24 h windows.

If sensors are calibrated in such environments, mappings with this nature will be generated, a consequence that was already hypothesized by De Vito et al. [24]. Figure 4a reveals how overlapping the standardized time traces of the reference signals can be, which appears not to be the case for sensor signals (Figure 4b). Models learn to describe and reproduce a process which is local, but other processes with different stoichiometry exist as well. Since the aim is not to model the environment but to calibrate sensors, this is a sampling bias and as such related to data representativeness. In statistics, this is a bias in which instances are collected so that some members of the natural population have a lower or higher sampling probability than others—mostly known from social sciences. ML is the toolbox to compress the content of data to rules such as decision boundaries or regression lines, and with this in mind, it is only reasonable that field calibrated models summarize meteorological conditions and their relationship with each other. Ideally, there should be no intrinsic pattern in the data, i.e., the sampling space should be unbiased. In this manner, an algorithm can learn properly which sensors are influenced by which outputs or environmental factors.

To provide a concrete example, suppose a low-cost sensor is affected by its target gas species, but also by T and AH. However, in the hypothetical data for calibration, the gas species, T, and AH increase and decrease jointly; hence, the variables are correlated. Consequently, it is not possible to learn the individual contributions of the three variables to the sensor signal, since they are always superimposed. (Moreover, it might be even possible to “measure” the target gas compound with a sensor for T or AH.) In statistics, the theory of experimental design has been developed specifically for this purpose, i.e., to avoid such confounding while minimizing the number of performed experiments [23].

Another issue with field calibration is temporal variation of the atmospheric state, i.e., non-stationary joint probability distribution of output variables and environmental conditions. More precisely, a sensor calibrated with a time series of a few days (training phase) might not be able to capture ground level concentrations in the following weeks or months if said distribution, i.e., the combinations of observable output variables and environmental conditions and their relationships, changes significantly during test phase. In the original publication, De Vito et al. claimed that two weeks of training data would be sufficient (far less than the two months computed by the learning curve), which seems feasible as relationships between references appear to be stable over time in this particular case [24]. More precisely, Figure 4a shows how several time intervals contain slightly less variation than others, but the correlations between references are persistent.

In Figure S8a, a PC analysis of the standardized reference data, combined with the sensor signal of T and AH, with 200 days of hourly measurements colored in slices of ten days is visualized, revealing that some parts of the data are dissimilar to others. Furthermore, the collection of points is neither completely overlapping nor symmetric, which would be better suited for calibration (Figure S8b). PC 1 is mainly composed of the reference data and explains a variance ratio of 0.60 (Figure S9a), whereas PC 2 is composed of T and AH sensor information and explains a variance ratio of 0.25 (Figure S9a). For calibration, every PC should be composed of only one variable and all PCs should explain an equal ratio of variance (uncorrelated and standardized reference data). Since variation occurs along PC 2, it can be concluded that T and AH conditions are indeed evolving with time, a detail that is also supported by Figure 4b, leading to conditions at which sensors have not been calibrated or tested, which could potentially cause problems over the course of a test phase. Moreover, it might be hard to estimate the contribution of drift due to aging in a changing environment under the assumption of interferences with T and/or AH.

Using a short time interval for calibration in a case where relationships are evolving over time would render the model useless within weeks, since it is of high importance that training and test data sets are comparable, a fact that is also recognized by De Vito et al. [24]. In those scenarios, a longer time series might be desirable to capture the complete joint probability distribution of sensor signals, reference values, and environmental conditions but drift due to sensor aging, i.e., the need for recalibration, makes this approach unappealing so far. Ideally, the whole feature space should be sampled in a more systematic manner to make the calibration robust, which is usually only possible in laboratory environments including some design of experiments. Alternatively, such data could be used complementary to compensate for unobserved combinations between input/output variables. However, this approach can be more expensive because many data points might be necessary to apply ML methods; Bigi et al. have presented non-linear and non-monotonic partial dependences between input and output variables, which are only discovered with many different combinations of pollutants and environmental conditions, if assumed to be true [9]. Due to sensor aging, regular calibration intervals would be needed, and together with the aforementioned aspects, an automated, large-scale calibration approach would be desired to make this all affordable.

It could be of interest to inspect other data sets that have been used in similar studies; instead of repeating the analysis several times, it is sufficient to examine the global structure of the collected reference data. For instance, Zimmermann et al. measured several gas compounds with low-cost sensors and reference instruments in the proximity of a small-sized parking lot; the reference data are mostly complete from the third month on, whereas 90 days of quarter-hourly measurements are analyzed starting from this month [30]. In Figure S10a, the Spearman rank correlation matrix is depicted, and it shows moderate correlations between the reference gases. Furthermore, there is some drift of the distribution over time, as seen in Figure S10b. The first two PCs explain a variance ratio of about 0.70 (Figure S10c,d); hence, there is a pronounced interdependence of the references and environmental conditions.

In a similar field study performed by Spinelle et al., data from a semi-rural site was acquired over a period of three months using low-cost sensors and gas analyzers [31]. In Figure S11a–d, the same plots are drawn as above, and the overall situation appears just slightly less problematic with respect to correlations on first sight, but the first two PCs again explain about a fraction of 0.62 in variance. Spinelle et al. recognized that this was a problem, and they write about (Pearson) correlations, too: “This […] shows that the dataset suffers from an important lack of independence between parameters. As example, CO_2_ show a high negative correlation with temperature … and a high positive correlation with relative humidity … Although, it is well known that temperature and humidity are important factors that may affect sensors responses. Using only field tests with uncontrolled temperature and humidity conditions makes impossible the distinction between the temperature and humidity effects on the sensor response”. They acknowledged correctly that the decomposition of the individual interfering effects is not possible. (Moreover, the fact that correlations, i.e., relationships, between molecules are different in the three locations indicates that generalization of calibrations models developed from field data is limited).

To make the bridge between metrology and social sciences, it is worth mentioning briefly that the relocation problems are, in fact, not self-contained but related to the discussion of fairness and representativeness of data in artificial intelligence (AI). The discussion around bias in AI, in which ML is part of, is an old one and it has rightly made its way into metrology [32,33]. Additionally known under the name “algorithmic bias”, it revolves around human bias (which is often present in data) that propagates into AI-based systems. One fictitious example from human resources would affect the hiring process; since more executives are male, more of them might be labelled as “qualified” (in a corporate data set) for a management role in comparison to their female colleagues [34]. A female applicant might find herself discriminated by an AI-system that automatically pools candidates for open leadership positions just because skewed or biased data are used during model development.

In data science scenarios, in which a lot of data are put into one pot to be distilled, tracking these cases becomes incredibly difficult. In the presented study, the demonstrated sampling bias was only uncovered by exploring the models and data with model inspection techniques, correlation matrices, histograms, and so on. This is one of the reasons why explainable AI is highly anticipated, e.g., models explaining their predictions and making clear how specific decisions have been made [35,36]. However, many top-performing speech and image recognition models are black-boxes based on NNs [37].

Researchers and institutions developing products embedded with AI, e.g., automated decision-making, should be made responsible for guaranteeing that nobody is discriminated (or life put at risk due to low air quality) but currently there is no regulation at all. Ideally, there will be an independent and credible organization that certifies and assesses uncertainty of such products in the future. In AQM using low-cost devices with built-in gas and/or PM sensors, there is the possibility of an undetected health risk for people, which is why improper calibration has to be avoided. For those applications, metrology institutes can fulfill this role by offering standardized data generation procedures for calibration to reduce bias as much as possible. Moreover, to assess air quality, monitoring devices have to be accurate in the sense that uncertainty of their measurement results has to be determined according to internationally agreed methods [38]. Only with dedicated characterization procedures can it be guaranteed that this uncertainty lies below the data quality objective as defined in the EU air quality directive, e.g., 25% uncertainty on the measurement (95% confidence interval) for NO_2_ [39].

Generating representative data and with as little bias as possible for ML models is not trivial due to the need for infrastructure and professional competence to synthesize reference gas mixtures and/or aerosols with different compositions and under varying environmental conditions (i.e., T, P, and AH) inside climate chambers. Nevertheless, metrology institutes have the capabilities to generate references that are traceable to the international system of unit and internationally recognized [40,41].

More precisely, gas mixtures with varying amounts of substance fraction, i.e., in nmol/mol (ppb) or µmol/mol (ppm), in the range of atmospheric concentrations can be synthesized using different chemical principles according to the compound(s) of interest [42]. For instance, O_3_ is generated with a reference standard photometer at the ppb level [43]. NO and CO are produced with gravimetric gas standards and dynamic dilution at the ppb and ppm level, respectively [44]. Lastly, NO_2_ is generated with a dynamic permeation process at the ppb level [45,46,47]. Additionally, flows are calibrated with a primary volumeter, which allows a well-defined multi-component mixture associated with its uncertainty [48,49].

In comparison to field calibration, the described laboratory techniques are capable of generating representative data, but they are also more time-consuming and infrastructure-demanding. It is evident that all this comes at a higher price, but the advantage of metrology institutes is to have such an infrastructure almost fit-for-purpose, which could be upgraded and optimized in the future to lower cost of data generation, e.g., via automatization. In addition, investing in the improvement of test chambers/benches to enable parallel calibration of multiple low-cost devices could decrease the cost of laboratory calibration even further.

## 4. Conclusions and Outlook

Low-cost AQM systems seem appealing but the need for thorough engineering and frequent (re)calibration pose a serious problem. Collocation to reference stations, i.e., field calibration, is believed to partially overcome some of these flaws. Despite some studies in which field calibration has apparently been applied successfully, this approach should only be followed with caution since correlations between pollutants and non-uniform sampling can lead to calibration functions susceptible to spatial or temporal relocation independent of the applied algorithm, as undesired relationships between measured variables might be integrated into models. Every model is only as good as the data it has been trained with. Generating this data in laboratory environments would be desirable, but this approach has to be scalable and become automated in order to be affordable. Only in this manner relationships between ground level concentrations, environmental conditions, and sensors could be learned reliably with as little bias as possible, which is left to prove for future research.

## Figures and Tables

**Table 1:** Optimal hyperparameters (HP_opt_) and model performance on test set data. The differences in performance can be considered negligible.

	NN	RF	LR
HP_opt_	1.60	12	0.03
MSE	0.10	0.09	0.10
R^2^	0.83	0.86	0.85
***ρ***	0.81	0.83	0.84

## Supplementary Materials

Supplementary figures including the results for RF and LR algorithms are available in a separate document at https://www.mdpi.com/1424-8220/20/21/6198/s1, Figure S1: (a) Learning curve for training (blue) and validation (green) data sets for final RF model (with mean and standard deviation); validation set performance increases with increasing sample size N; (b) Validation curve for training (blue) and validation (green) data sets for final RF model (with mean and standard deviation); validation set performance increases with deeper trees and flattens above a maximum depth of 9; Figure S2: (a) Learning curve for training (blue) and validation (green) data sets for final LR model (with mean and standard deviation); validation set performance increases with increasing sample size N and flattens after 1800 instances; (b) Validation curve for training (blue) and validation (green) data sets for final LR model (with mean and standard deviation); validation set performance increases with decreasing α but is mostly flat below α = 100; Figure S3: (a) Model performance of RF with respect to individual references; (b) Permutation importance of all features in RF model; Figure S4: (a) Model performance of LR with respect to individual references; (b) Permutation importance of ten most important features in LR model; Figure S5: Partial dependence of references on sensors in the NN model; Figure S6: Partial dependence of references on sensors in the RF model; Figure S7: Partial dependence of references on sensors in the LR model (six most important features); Figure S8: (a) PC analysis of standardized reference signals combined with T and AH sensor signals. Each color represents a slice of ten days with 20 plotted slices in total, and it can be seen that some parts of the data are different from others as the collection of points is not circular and not completely overlapping; (b) PC analysis of synthetic data under the hypothesis of independence and normal distribution; Figure S9: (a) Loadings of PC 1 (explained variance ratio of 0.60), which is mainly composed of the reference data; (b) Loadings of PC 2 (explained variance ratio of 0.25), which is mainly composed of T and AH sensor signals; Figure S10: (a) Spearman rank correlation matrix of sensor (s) and reference (r) signals; (b) PC analysis of standardized reference signals combined with T and AH sensor signals; (c) Loadings of PC 1 (explained variance ratio of 0.49); (d) Loadings of PC 2 (explained variance ratio of 0.22); Figure S11: (a) Spearman rank correlation matrix of reference (r) signals; (b) PC analysis of standardized reference signals; (c) Loadings of PC 1 (explained variance ratio of 0.44); (d) Loadings of PC 2 (explained variance ratio of 0.18). The code developed in the analysis can be found on GitHub at https://github.com/gtancev/Chemical-Sensor-Data.
