# Supplementary material for: The Relocation Problem of Field Calibrated Low-Cost Sensor Systems in Air Quality Monitoring: A Sampling Bias
Source: Sensors (Basel). 2020 Oct 30;20(21):6198. doi: 10.3390/s20216198 (PMC7662848; doi:10.3390/s20216198)
Supplement: Supplementary file 1 [file sensors-20-06198-s001.pdf]

Supplementary Material

The Relocation Problem of Field Calibrated Low-Cost Sensor Systems in Air Quality Monitoring: A Sampling Bias

Georgi Tancev * and Céline Pascale

Swiss Federal Institute of Metrology, 3084 Bern, Switzerland; celine.pascale@metas.ch
* Correspondence: georgi.tancev@metas.ch

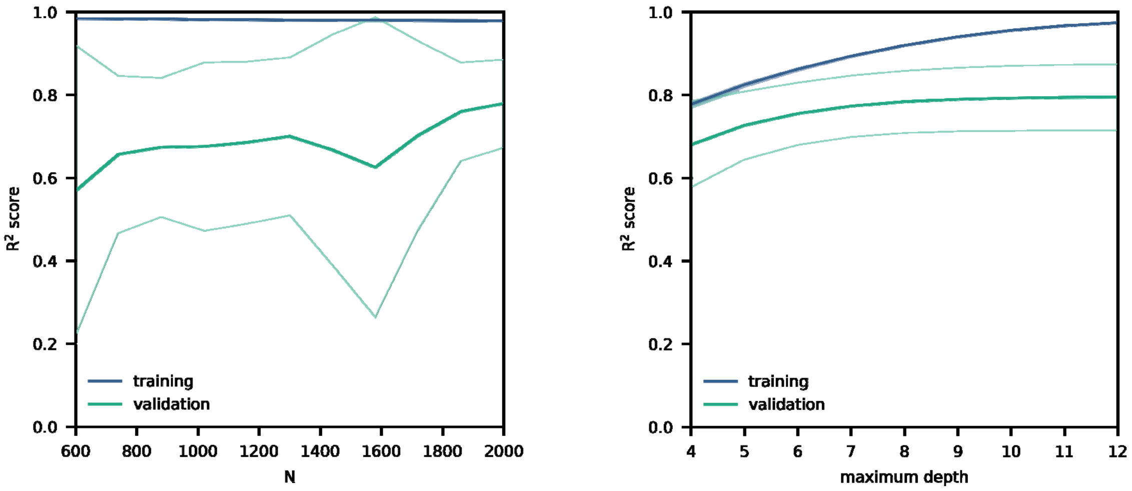

(**a**) (**b**)

Figure S1. (a) Learning curve for training (blue) and validation (green) data sets for final RF model (with mean and standard deviation); validation set performance increases with increasing sample size N; **(b)** Validation curve for training (blue) and validation (green) data sets for final RF model (with mean and standard deviation); validation set performance increases with deeper trees and flattens above a maximum depth of 9.

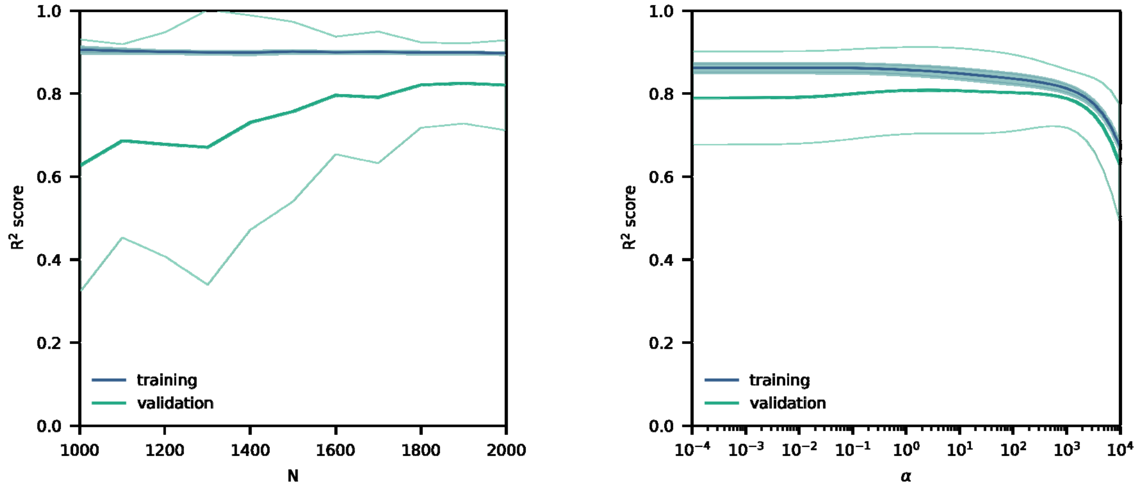

(a) (b)

Figure S2: (a) Learning curve for training (blue) and validation (green) data sets for final LR model (with mean and standard deviation); validation set performance increases with increasing sample size N and flattens after 1800 instances; **(b)** Validation curve for training (blue) and validation (green) data sets for final LR model (with mean and standard deviation); validation set performance increases with decreasing α but is mostly flat below $\alpha = 100$.

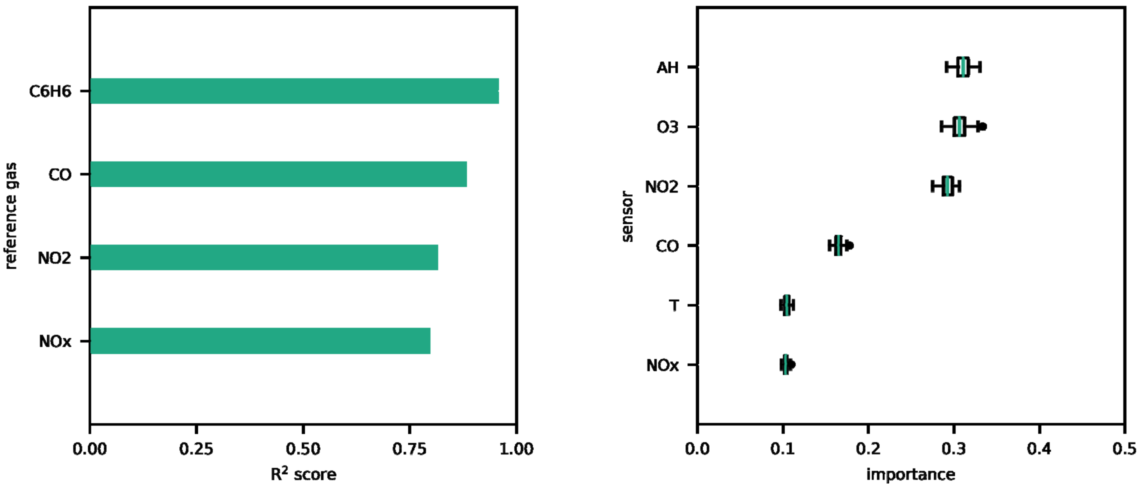

(a) (b)

Figure S3: (a) Model performance of RF with respect to individual references; **(b)** Permutation importance of all features in RF model.

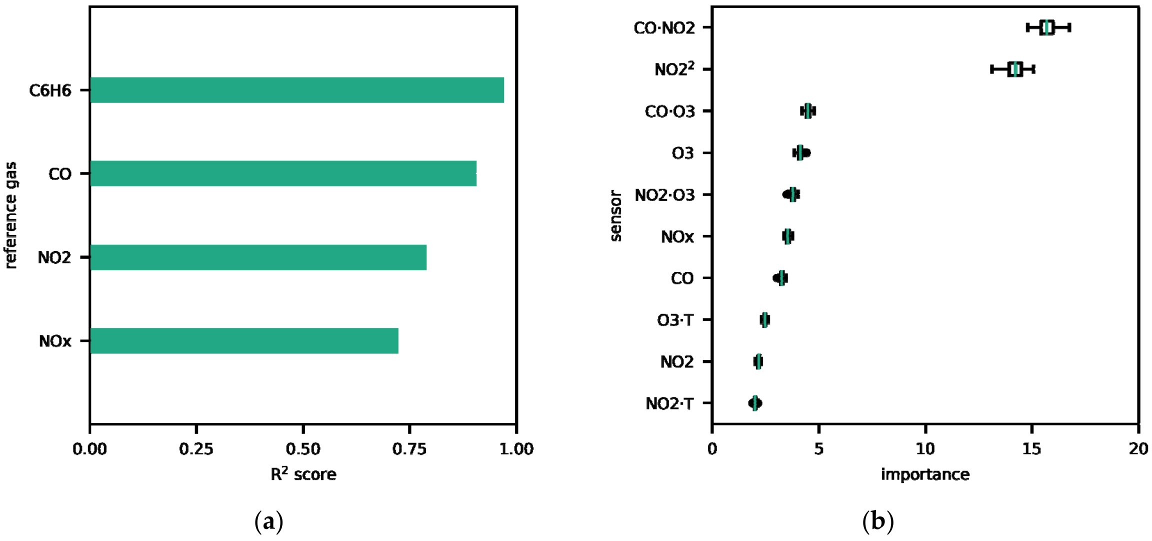

Figure S4: (a) Model performance of LR with respect to individual references; **(b)** Permutation importance of ten most important features in LR model.

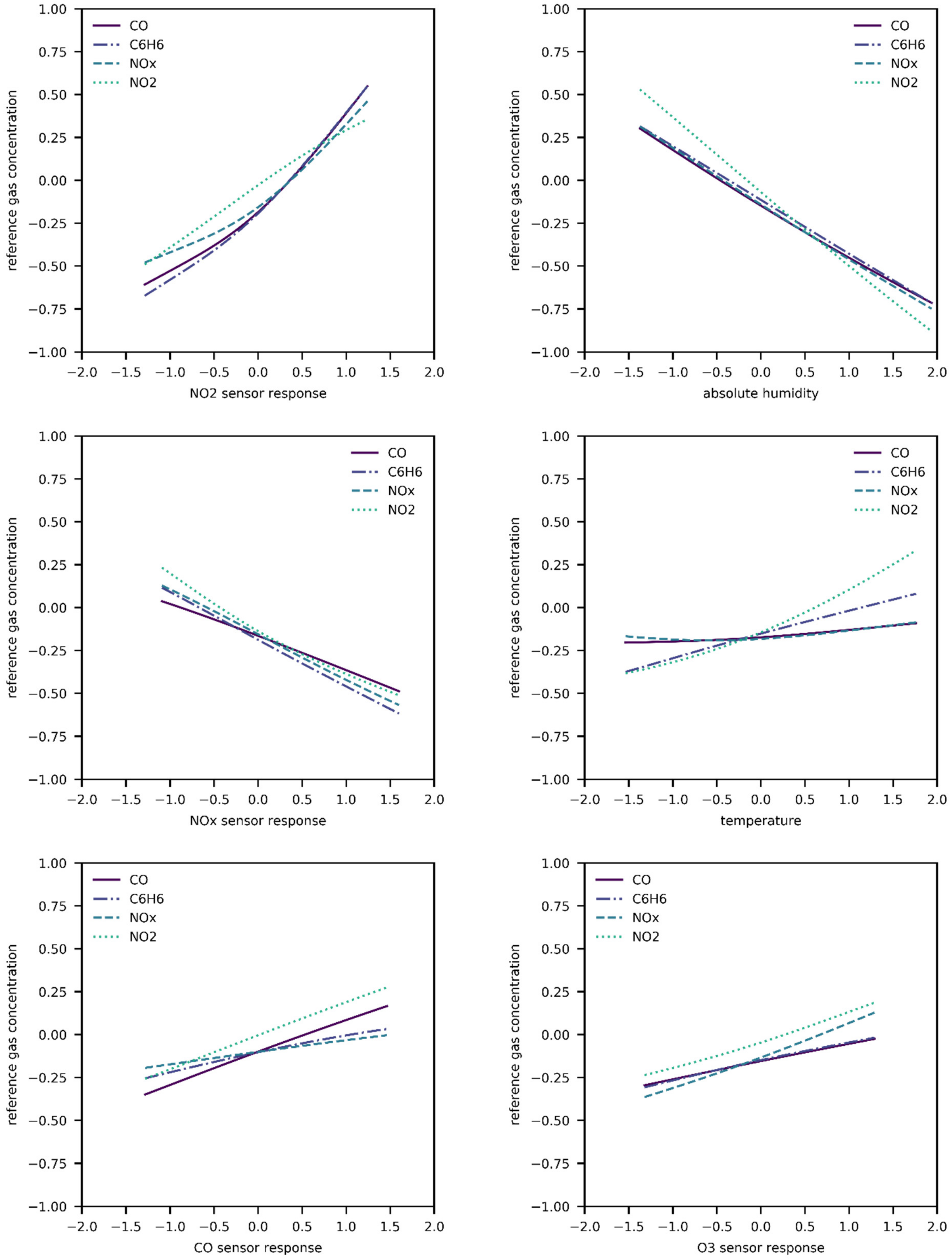

Figure S5: Partial dependence of references on sensors in the NN model.

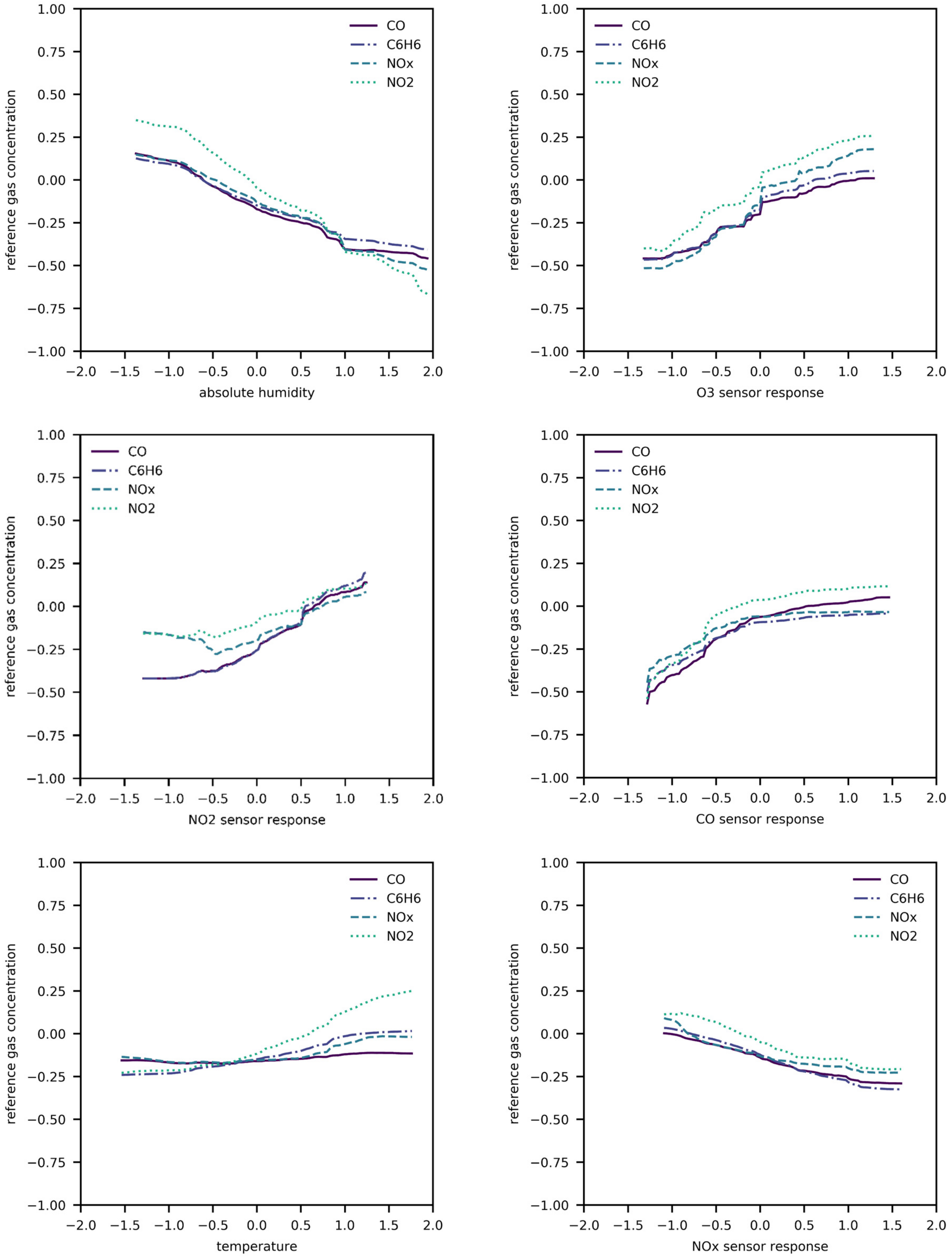

Figure S6: Partial dependence of references on sensors in the RF model.

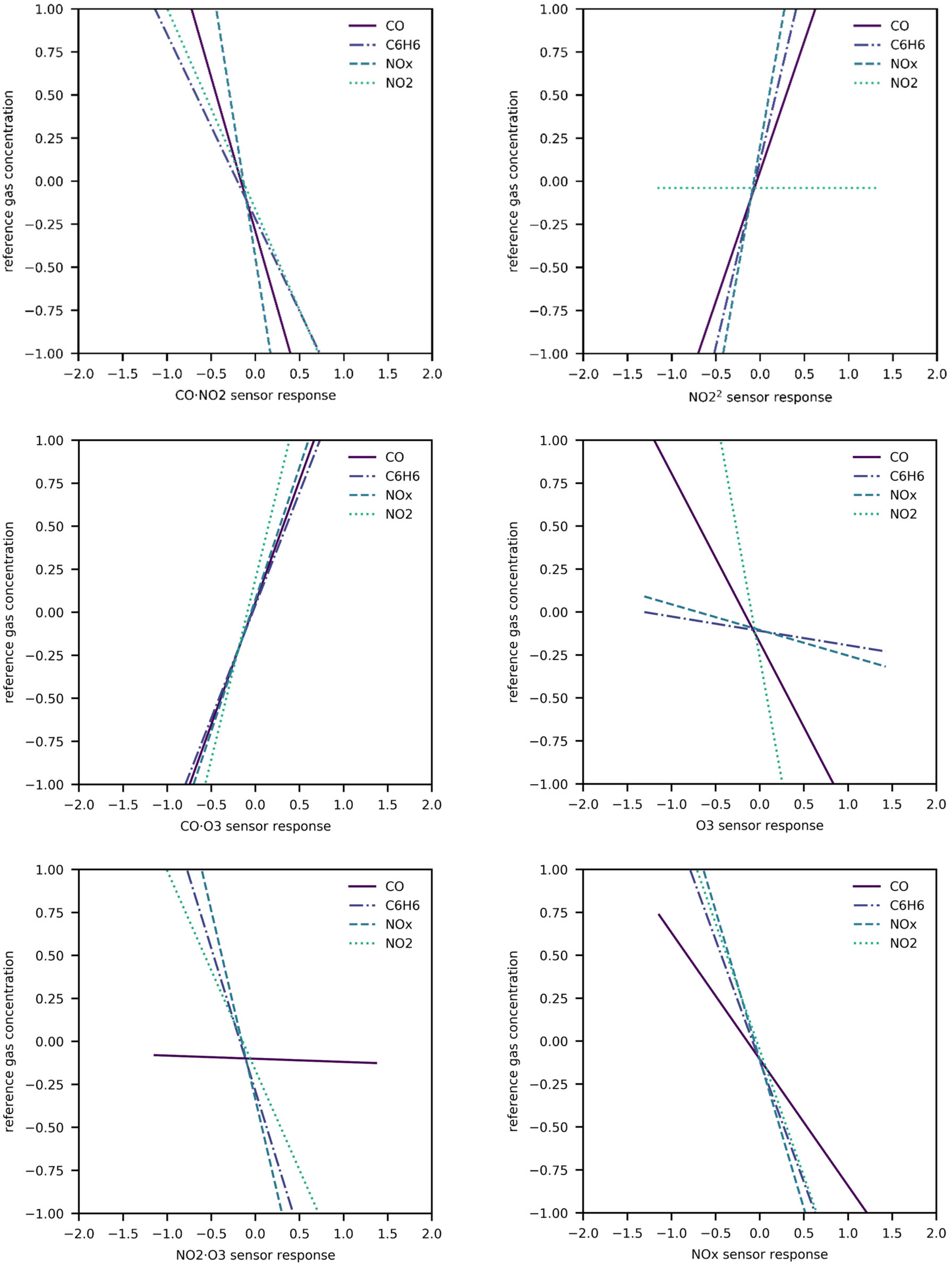

Figure S7: Partial dependence of references on sensors in the LR model (six most important features).

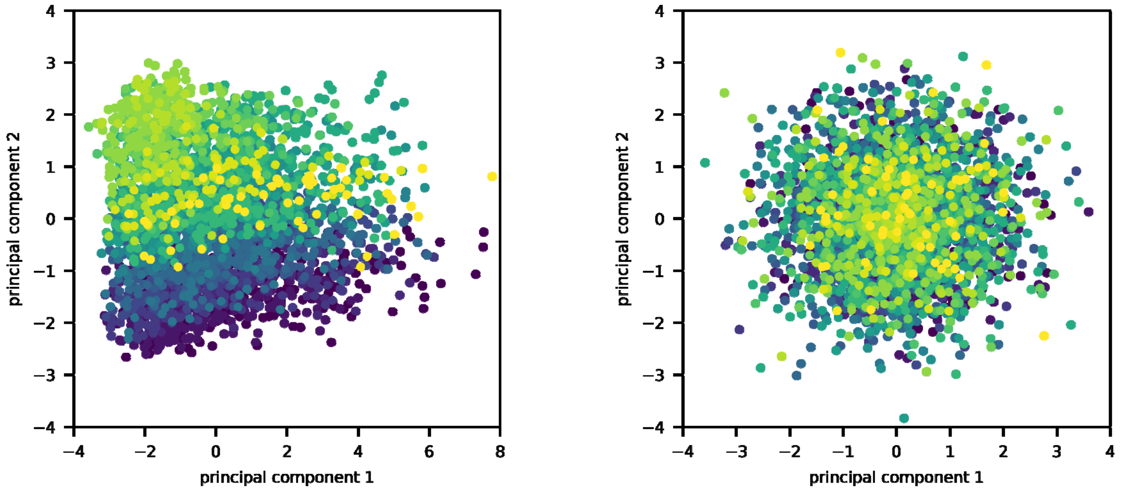

(**a**) (**b**)

Figure S8: (a) PC analysis of standardized reference signals combined with T and AH sensor signals. Each color represents a slice of ten days with 20 plotted slices in total, and it can be seen that some parts of the data are different from others as the collection of points is not circular and not completely overlapping; **(b)** PC analysis of synthetic data under the hypothesis of independence and normal distribution.

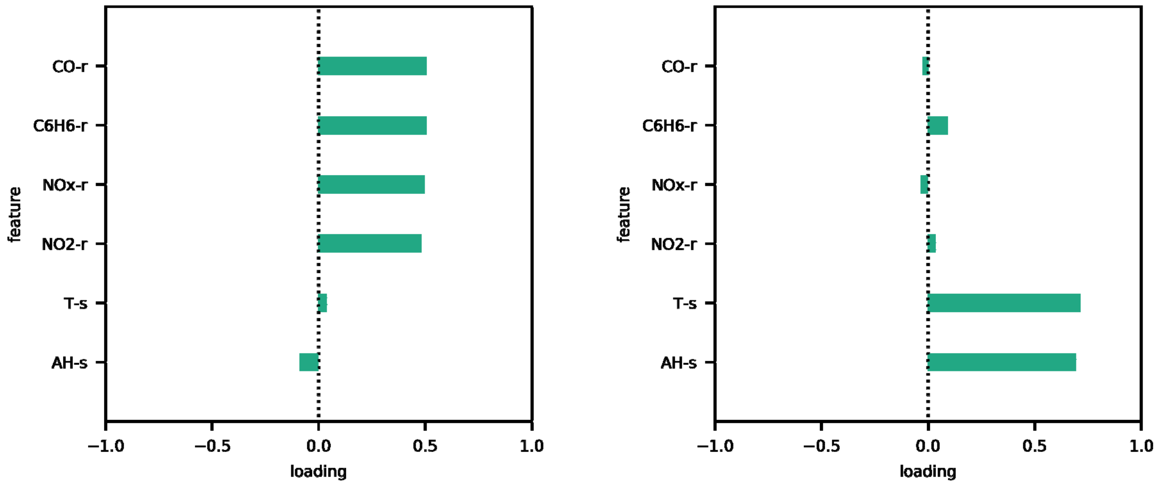

(**a**) (**b**)

Figure S9: (a) Loadings of PC 1 (explained variance ratio of 0.60), which is mainly composed of the reference data; **(b)** Loadings of PC 2 (explained variance ratio of 0.25), which is mainly composed of T and AH sensor signals.

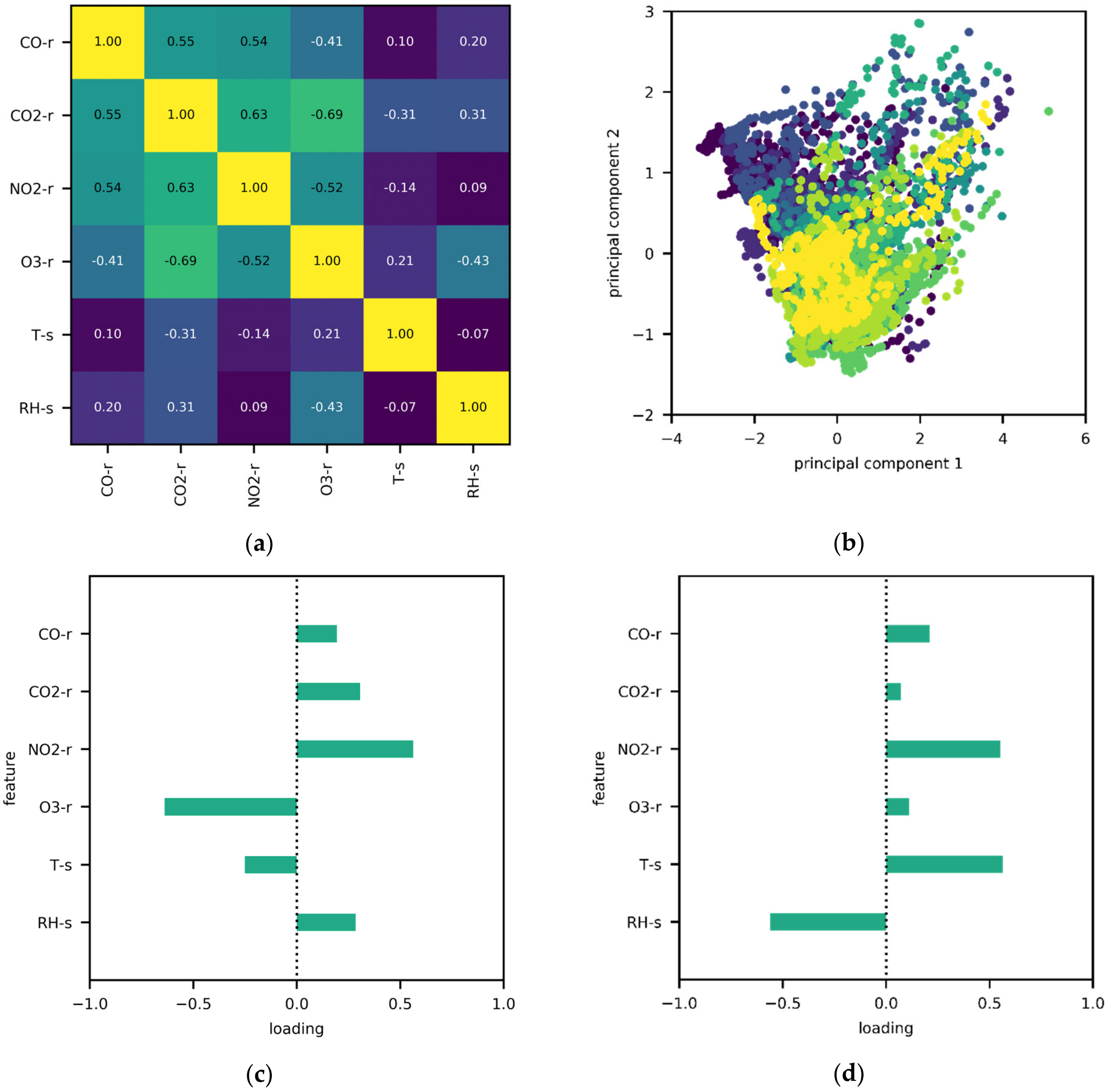

Figure S10: (a) Spearman rank correlation matrix of sensor (s) and reference (r) signals; **(b)** PC analysis of standardized reference signals combined with T and AH sensor signals; **(c)** Loadings of PC 1 (explained variance ratio of 0.49); **(d)** Loadings of PC 2 (explained variance ratio of 0.22).

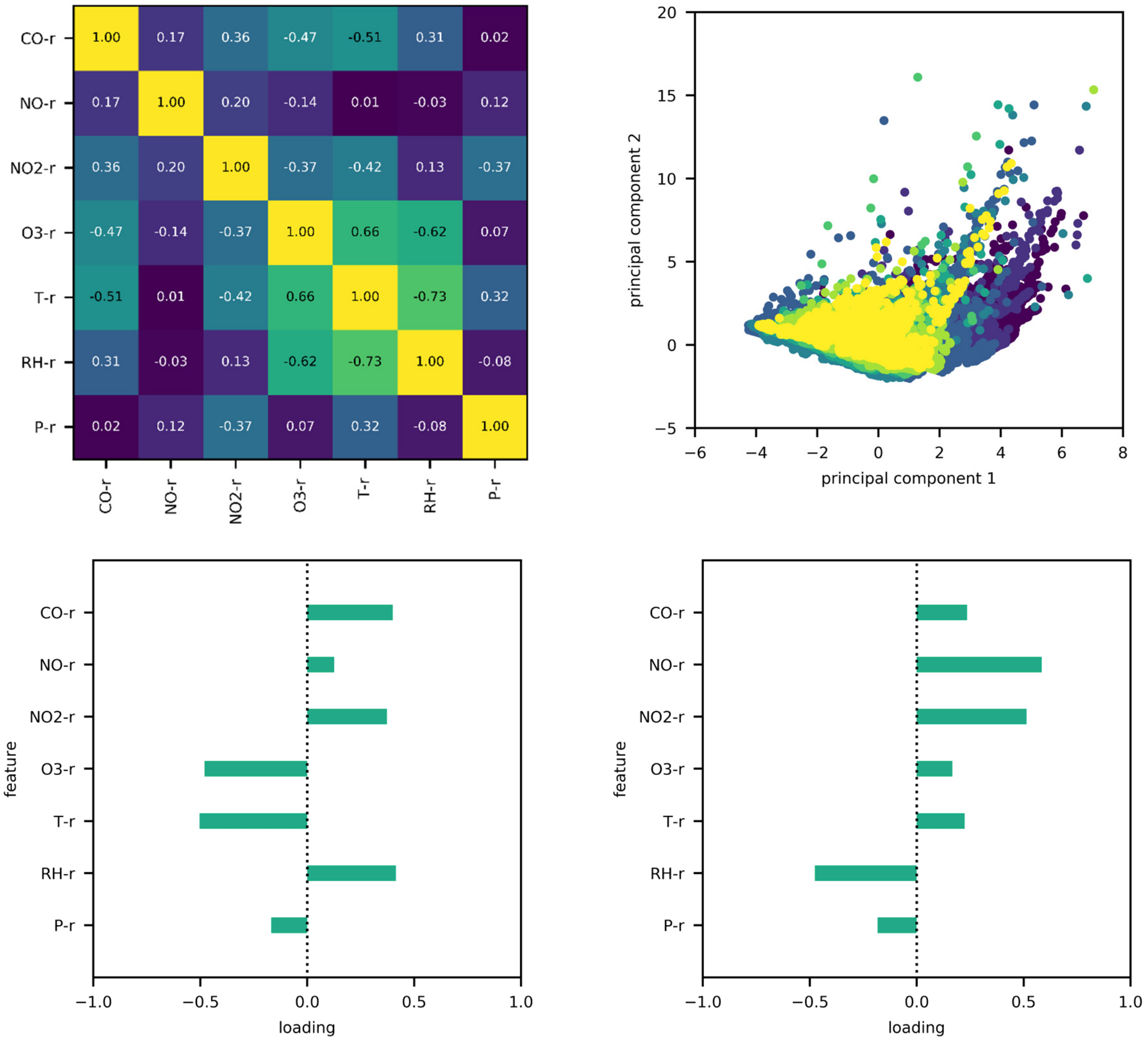

Figure S11: **(a)** Spearman rank correlation matrix of reference (r) signals; **(b)** PC analysis of standardized reference signals; **(c)** Loadings of PC 1 (explained variance ratio of 0.44); **(d)** Loadings of PC 2 (explained variance ratio of 0.18).
